# Exercise Dependence and Anxiety in Cross-Trainers, Bodybuilders and Gym Exercisers During COVID19

**DOI:** 10.1177/00315125221098326

**Published:** 2022-05-16

**Authors:** Rogério Salvador, Roberta Frontini, Catarina Ramos, Pedro Lopes, Janete Oliveira, Joana Maia, Diogo Monteiro

**Affiliations:** 1 CIEQV - Life Quality Research Centre, 70867 Polytechnic of Leiria, Leiria, Portugal; 2 ESECS - 70867 Polytechnic of Leiria, Leiria, Portugal; 3 Center for Innovative Care and Health Technology (ciTechCare), 70867 Polytechnic of Leiria, Leiria, Portugal; 4 Research Center in Sports Sciences, Health Sciences and Human Development (CIDESD), Universidade de Trás-os-Montes e Alto Douro (UTAD), Vila Real, Portugal

**Keywords:** COVID19, exercise dependence, anxiety, gym practitioners, cross training

## Abstract

The World Health Organization declared the COVID-19 pandemic an international public health emergency in January 2020, and, soon thereafter, a worldwide adoption of quarantine and physical isolation measures restricted regular practitioners of indoor group physical exercise from many of their usual practices. Some, with exercise dependence (ED), may have experienced exercise withdrawal symptoms that triggered unhealthy anxiety levels. In February 2021, during Portugal’s second COVID-19 lockdown, we characterized and compared ED and anxiety levels among different groups of indoor exercise practitioners (cross trainers [CG], bodybuilders [BG] and gym practitioners [GG]). In this cross-sectional study, we recruited 234 adult participants through the internet. To assess participants’ ED and anxiety levels, we used Portuguese versions of the ED Scale-21 (EDS-21) and the State-Trait Anxiety Inventory (STAI-State; STAI-Trait). ED symptoms were evident in all participant subgroups, and we found no gender differences in ED. Anxiety was higher among women than men in CG and GG groups, and there were significant differences in ED between groups such that BG practitioners showed higher ED than GG and CG practitioners (small effect size). Bodybuilders reported most ED behavior, followed by CG and regular gym exercisers, but on some criteria BG and CG groups had similar ED levels. Our results are in line with prior ED prevalence reports conducted before COVID-19 restrictions among regular GG, but these are the first data to report a higher ED prevalence among BG and CG, relative to GG.

## Introduction

The COVID-19 outbreak was declared a public health emergency of international concern by the World Health Organization (WHO) on January 30, 2020 (World Health Organization, 2020a); and, on 11 April 2020, the WHO declared COVID-19 a pandemic (World Health Organization, 2020b). In Portugal, a state of emergency was decreed on March 18, 2020, when Portugal implemented “… *extraordinary and urgent measures to restrict rights and freedoms, especially with regard to the rights of free movement and economic freedoms, in concert with European authorities to prevent the virus transmission*” (President of the Republic Decree no. 14-A/2020). This implied a national adoption of measures of quarantine and physical isolation. These measures may have protected public health by preventing and/or mitigating virus transmission, but studies have shown several negative psychological effects of social isolation that include high levels of anxiety, stress, fear, or even depressive symptoms that can persist beyond the period of social restraint (e.g., Antunes et al., 2020). One of the changes in people’s daily routines during these recent periods of quarantine and isolation was a restriction from the use gyms and other group sports facilities. Many regular practitioners of indoor physical exercise were unable to carry out their usual practices and experienced a change in their routines that might have precipitated withdrawal symptoms for those with an exercise addiction, making an assessment of indoor exercise practitioners’ behaviors and feelings during this unusual and stressful time important.

Physical exercise has been defined as a structured and planned action that promotes physical and mental health (Dasso, 2019), and its practice has been recommended by several reference institutions (U.S. Department of Health and Human Services, 2018; World Health Organization, 2018). However, past research has also shown that many individuals may have a less than healthy relationship with physical exercise, such that these activities can be associated with the development of disruptive behaviors and with their own symptoms of physical, mental, and social difficulties (Alcaraz-Ibañez et al., 2021). When practitioners develop an excessive focused on exercise goals or when exercise behaviors interfere with the practitioners’ personal, professional, and social lives, high levels of depression and anxiety can result (Weinstein et al., 2017; Egorov & Szabo, 2013; Alcaraz-Ibañez et al., 2021). There may even be a type of addictive exercise behavior involving compulsive exercise or exercise dependence (ED) (Voelker et al., 2015) that is often associated with such other disorders as body image dissatisfaction, weight loss, eating disorders, and as mentioned, unhealthy anxiety. We can define ED as addictive exercise behavior in which the individual manifests behaviors and symptoms seen with other addictions, such as abstinence syndrome, loss of behavioral control, excessive time dedicated to the activity and a disturbance of mood and tolerance (Marques et al., 2019).

Different authors attempting to understand this exercise-related addictive behavior have reported estimates of ED prevalence ranging from 3–7% for regular exercisers and university students and between 6–9% for athletes (Marques et al., 2019). In the field of physical exercise and fitness, there are various types of regular indoor physical exercise practice, including cross training and bodybuilding. These different forms of practice may correspond to different practitioner behaviors, partly as a function of the different objectives and motivations that lead people to these exercise choices. It is important to separate ED from gym enthusiasm; and those distinctions can be identified by whether, or not practitioners show symptoms of addictive tolerance, lack of control, and/or a decreased engagement in other activities (Berczik et al., 2012).

Studies comparing individuals who use and do not use gyms for exercise have found that exercisers who do not use the gym, especially males, have tended to report less anxiety about their bodies than gym users. Stapleton et al. (2016) specifically related eating disorders, due to appearance concerns, to male gym users. There has been some evidence that most gym users seek gains in muscle mass, but it is not clear whether this trend would be more evident among bodybuilders than recreational gym users who do not necessarily seek a “performance physique” (Stapleton et al., 2016). Exercise dependence is not yet considered a form of addiction within the Diagnostic and Statistical Manual of Mental Disorders (DSM-5; American Psychiatric Association, 2013), but ED constitutes a potential public health problem, as it often presents a bidirectional relationship with other addictions and with disruptive behaviors. Thus, it is relevant to further investigate ED in the context of similar behavioral models and to produce knowledge that will allow clinicians and researchers to better understand when or if preventive and/or intervention strategies are needed for unhealthy ED.

Since COVID-19-induced social distancing requirements may have precipitated particular anxiety for some exercisers using indoor facilities, we aimed, in the present study, to characterize and report levels of ED and anxiety in different groups of gym practitioners during the COVID-19 pandemic. We hypothesized that practitioners of cross training would show a higher prevalence of ED symptoms than practitioners of gym activities but a lower prevalence of ED symptoms than bodybuilders. We also expected males to show a higher prevalence of ED symptoms than females. Other studies have reported similar ED prevalence values among cross trainers and regular exercise practitioners (Lichtenstein & Jensen, 2016), but there have been few replication studies of this observation, and most studies have been carried out exclusively with males, leaving minimal gender comparison data (Marques et al., 2019).

## Method

Our research design was a cross-sectional comparison of male and female exercisers from several different indoor exercise practice groups (i.e., cross training, body building and a group of other gym activities, consisting of cardio training, resistance training and group activities). We conducted this research through online surveys in Portugal, administered between 2-17 February 2021, when Portugal was in a second COVID-19 lockdown.

### Participants

Our participant sample consisted of 234 adults (133 women, 101 men; *M*_age_ = 32.5, *SD* =11.27 years) who were recruited online. We used Facebook and Instagram social media to advertise and recruit participants who received no compensation for their participation, and we used Google Forms as the survey platform for electronic distribution. The study was conducted following the Declaration of Helsinki guidelines for the treatment of human participants in research (World Medical Association, 2013). We obtained ethical approval from the Committee of the Quality-of-Life Research Centre (CIEQV) under the reference UIDB/04748/2020. Participants provided their informed consent online before completing the surveys, and their anonymity was assured. Participants understood that they could withdraw from the study at any time.

The assessment protocol consisted of self-reported questionnaires assessing different domains of everyone’s behavior and feelings (see a full description of these measures below). Inclusion criteria were: (a) involvement in cross training, bodybuilding, or gym activities (cardio training, resistance training and group activities; (b) three months of continuous practice in these exercises before a second Portuguese lockdown was declared in January 2021 at gyms or fitness and sports facilities. Participants were divided into three groups of practice: (a) a gym group (GG) comprised of various gym activities like cardio training, resistance training and group activities (72 women, 29 men; *M*_age_ = 34.7 years, *SD* =13.42); (b) a cross training group (CG) who engaged only in cross training or CrossFit activities (35 women, 49 men; *M*_age_ = 34.2, *SD* =7.56 years); and (c) a bodybuilding group (BG) who only engaged in bodybuilding training (26 women, 23 men; *M*_age_ = 25.0, *SD =*8.40 years).

### Assessment Measures

Our participant characteristic questionnaires assessed three domains: sociodemographic and personal data (i.e., gender, age, height, weight, education level, and practice experience), ED or exercise dependence, and anxiety (state and trait anxiety). The survey with sociodemographic questions was previously developed and reviewed by specialists in exercise and psychology.

### Exercise Dependence

To assess ED we used the Portuguese version (Lindwall & Palmeira, 2009) of the ED Scale-21 (EDS-21). This is a 21-item, multidimensional measure of ED, based on criteria within the DSM-IV (American Psychiatric Association, 2013) for substance dependence, including “Tolerance” (i.g., a need for increasing amounts of exercise to achieve the desired results or diminishing effects from the same amount of exercise), “Withdrawal” (i.g., symptoms of withdrawal from the exercise or the same amount of exercise is undertaken to relieve or avoid withdrawal symptoms), “Intention effects” (i.g., often engaging in more exercise than planned), “Lack of control” (i.g., a persistent desire or unsuccessful effort to cut down or control exercise), “Time” (i.g., spending too much time in exercise-related activities), “Reduction in other activities” (i.g., occupational, social, or recreational activities are reduced or given up because of exercise), and “Continuance” (i.g., exercising despite injury or illness) (Smith et al., 2010). The EDS-21 establishes cut-off criteria to distinguish individuals who are at risk for ED, as compared to those who have some or no ED symptoms. The EDS-21 is scored on a 6-point Likert scale. In our study, it presented high internal consistency (α = .90).

### Anxiety

To assess participants’ self-reported anxiety, we used the Portuguese version (Silva, 2003) of the State-Trait Anxiety Inventory (Spielberger et al., 1983). This survey is comprised two forms (Form 1 and Form 2) with 20 statements each, evaluated on a 4-point Likert scale. Form 1-STAI-State evaluates transient or temporary anxiety, (i.e., the anxiety that the person is feeling at the moment), and Form 2-STAI-Trait assesses dispositional or general anxiety. The score is generated by summing the scores on the 20 items for each scale. Higher scores indicate higher anxiety levels. Internal consistency among participants in this study was good (state α = .93; trait α = .93)

### Data Analysis

To perform data analysis, we used the Statistical Package for the Social Sciences (SPSS, version 27.0; IBM Corp, Armonk, NY, United States). We computed descriptive statistics for all sociodemographic and study variables, including frequency counts (and proportions), means (*M*), standard deviations (*SD*), and 95% confidence interval (95% CI).

We performed independent samples *t*-tests (two-tailed) to assess the differences between participants’ gender (male vs. female). In addition, we used one-way analyses of variance (ANOVA) for comparisons of group differences. The ANOVAs were complemented with Bonferroni post-hoc tests to for pairwise comparisons, as necessary. Shapiro-Wilk (*n* < 50) and Levene´s tests were used to verify data normality and homoscedasticity, respectively. Cohen’s d (Cohen, 1988) analyses were performed to evaluate the effect size for comparisons between gender and partial eta-square was calculated to test the effect size across groups, as suggested by Ho (2014). The following cut-off values for effect size were assumed: “small” effect = .01, “medium” effect = .06, and “large” effect = .14; In addition, a chi-square analysis by group and gender was performed to analyze possible differences between groups and sex in ED classification prevalence. For all analyses we set the significance level to reject the null hypothesis at 5% (Ho, 2014).

## Results

### Participant Characteristics by Exercise Practice Group

The sociodemographic and personal characteristics of our participant sample are presented by their exercise practice group in Table 1. Regarding anthropometric measures, differences were found only in body weight between GG and CG (*F* (2, 231) = 4.67, *p* = .015; *n*^*2*^ = .039), with CG participants presenting a higher mean weight (*M* = 73.34, *SD =* 13.59 *kg*) than GG participants (*M* = 67.34, *SD* = 15.23 *kg*). Concerning participants’ educational levels, the most prevalent category of educational achievement in GG and CG groups was a college degree, with 53.5% and 41.7% of participants in these two groups achieving that level, respectively. A high school level of educational achievement was most prevalent among BG participants, with 53.1% of these participants reporting that educational level. In terms of practice experience, among GG and CG practitioners, the most prevalent report was 1–3 years of practice, with 29.7% and 39.9% reporting that prevalence, respectively. Among BG practitioners, the most prevalent practice experience report was more than three years, as reported by 53.1% of participants.

**Table 1.** Sample Sociodemographic and Personal Participant Characteristics by Practice Group (*n* = 234).

	Gym (*n* = 101)			Cross training (*n* = 84)			Bodybuilding (*n* = 49)		
	*n* (%)	Mean (*S*D)	(CI 95%)	*n* (%)	Mean (*SD*)	(CI 95%)	*n* (%)	Mean (*SD*)	(CI 95%)
Gender									
Female	72 (71.30)			35 (41.60)			26 (53.10)		
Male	29 (28.70)			49 (58.40)			23 (46.90)		
Age (years)		34.66 (13.42)	(18.00 - 77.00)		34.23 (7.56)	(22.00 - 51.00)		25.02 (8.40)	(18.00 - 48.00)
Height (cm)		167.08 (9.35)	(150.00 - 187.00)		170.07 (9.11)	(149.00 - 198.00)		171.82 (7.90)	(154.00 - 188.00)
Body weight (kg)		67.34 (15.23)	(44.00 - 145.00)		73.34 (13.59)	(50.00 - 100.00)		72.67 (13.47)	(51.00 - 104.00)
BMI (km/m^2^)		24.00 (4.36)	(16.73 - 45.76)		25.20 (3.29)	(19.53 - 34.89)		24.47 (3.38)	(17.65 - 35.22)
Education									
Basic education	3 (3.00)			3 (3.60)			-		
High school	32 (31.60)			31 (36.90)			26 (53.10)		
Degree	54 (53.50)			35 (41.70)			20 (40.80)		
Master’s degree	9 (8.90)			14 (16.70)			2 (4.10)		
Doctorate	3 (3.00)			1 (1.20)			1 (2.00)		
Practice experience									
3 to 6 months	28 (27.70)			17 (20.20)			10 (20.40)		
6 months to 1 year	15 (14.90)			9 (10.70)			2 (4.10)		
1 to 3 years	30 (29.70)			33 (39.30)			11 (22.40)		
More than 3 years	28 (27.70)			25 (29.80)			25 (53.10)		

*Note*: CI 95%, confidence interval 95%; *SD*: standard deviation*.*

### Within-Group Analysis

Independent sample two-tailed *t*-tests were performed to compare ED and anxiety levels within groups by gender, and these calculations are presented in Table 2. There were no significant gender differences on the Total EDS-21 score in any practice group. Regarding ED cut-off criteria, in the category of “Withdrawal effects” there were gender differences in GG (*t (99)* = −2.55, *p = .012*; *d* = .561) and CG (*t* (82) = −2.13*, p* = .036; *d* = .472), with higher mean withdrawal effects seen among females than males; there was a small effect size in both groups. Only participants in BG showed a significant gender difference on “Tolerance,” with the male group showing greater tolerance than the female group (*t* (47) = −2.47, *p* = .017; *d* = .707), with a large effect size. There was also a significant gender difference on “Lack of control” in GG (*t* (99) = 5.55, *p* = .020; *d* = .053), favoring males with a medium effect size. In the criteria of “Reduction in other activities,” “Time,” and “Continuance”, there were no significant gender differences in any practice group. We found a medium effect size and significant differences in “Interaction” between genders in the BG group, with males showing higher symptoms than females (*t = (47) = -2.16, p* = .036; *d* = .619).

**Table 2.** Comparison of Variables Between Gender and by Practice Group (*n* = 234).

	Gym						Cross training						Bodybuilding					
	Female (*n* = 72)		Male (*n* = 29)		t	d	Female (*n* = 35)		Male (*n* = 49)		t	d	Female (*n* = 26)		Male (*n* = 23)		t	d
	*n* (%)	Mean (SD)	*n* (%)	Mean (SD)			*n* (%)	Mean (SD)	*n* (%)	Mean (SD)			*n* (%)	Mean (SD)	*n* (%)	Mean (SD)		
ED criteria																		
Withdrawal effects		9.93 (4.15)		7.48 (4.85)	−2.55*	.561		10.49 (4.69)		8.83 (4.49)	−2.13*	.472		11.58 (4.87)		9.17 (4.73)	−1.75	.500
Continuance		6.63 (2.87)		6.52 (3.32)	−0.16	.036		8.11 (3.97)		8.22 (3.96)	0.13	.028		8.92 (4.68)		6.70 (3.52)	−1.86	.069
Tolerance		9.40 (4.33)		9.79 (3.94)	0.42	.092		11.34 (4.09)		11.78 (3.24)	0.54	.120		11.38 (3.93)		13.78 (2.64)	2.47*	.707
Lack of control		6.76 (3.40)		8.41 (2.55)	2.36*	.518		8.11 (4.15)		8.24 (3.64)	0.15	.034		7.96 (4.26)		8.43 (3.62)	0.42	.119
Reduction on other activities		5.90 (3.16)		6.14 (2.97)	0.34	.076		6.20 (2.47)		6.10 (3.05)	−0.16	.035		8.96 (3.59)		8.26 (4.04)	−0.64	.184
Time		7.92 (3.88)		7.90 (3.35)	−0.24	.005		8.86 (4.08)		8.39 (3.59)	−0.56	.123		11.65 (4.40)		11.04 (4.05)	−0.502	.144
Intention effects		6.00 (2.98)		6.62 (2.92)	0.95	.209		7.26 (3.39)		6.92 (2.96)	−0.49	.108		9.19 (3.45)		7.04 (3.49)	−2.16*	.619
EDS-21 total		52.54 (18.34)		52.86 (18.12)	0.80	.180		60.371 (9.49)		57.98 (16.35)	−0.61	.135		69.65 (19.25)		64.43 (17.93)	−0.98	.280
Classification for ED																		
Asymptomatic	30 (41.70)		8 (27.60)				4 (11.40)		9 (18.40)				4 (15.40)		3 (13.00)			
Symptomatic	38 (52.80)		20 (69.00)				26 (74.30)		37 (75.50)				15 (57.70)		18 (78.30)			
Dependent	4 (5.60)		1 (3.40)				5 (14.30)		3 (6.10)				7 (26.90)		2 (8.70)			
State anxiety		41.29 (11.10)		36.14 (9.40)	−2.20*	.483		45.49 (13.26)		35.67 (8.60)	−2.87**	.635		42.77 (10.59)		36.83 (11.14)	−1.91	.547
Trait anxiety		38.88 (9.98)		33.28 (8.71)	−2.64*	.581		37.71 (12.75)		32.80 (9.27)	−2.05*	.453		39.00 (9.11)		34.83 (10.73)	−1.47	.421

*Note*: CI 95%, confidence interval 95%; *SD* = standard deviation; ED = exercise dependence. *p < .05; **p < .01; ***p < .001.

While most participants of both sexes in all groups presented some ED symptoms (with ED symptom prevalence ranging between 52.8% (females in GG) and 78.3% (males in BG), the prevalence of an ED classification was much lower, ranging from 3.4% (males in GG) to 26.9% (females in BG). Of note, several prevalence levels appeared to be higher than have been previously reported among cross trainers and bodybuilders in prior research (Lichtenstein & Jensen, 2016; Soler et al., 2013). More specifically, this ED level was evident in 3.4% and 5.6% of males and females respectively in GG, 14.3% and 6.1% of females and males, respectively in CG, and 26.9% and 8.7% of females and males, respectively in BG. Females in all groups showed relatively high ED levels.

Regarding anxiety, gender comparisons were significant in GG and CG (but not BG) groups for both state and trait anxiety, with females presenting higher anxiety values compared to males. In GG the gender difference in state anxiety was of a small effect size (*t* (99) = −2.20, *p* = .030; *d* = .483); and, in CG, it was of a medium effect size (*t (82)* = −2.64; *p* = .036; *d* = .581). Regarding trait anxiety, we found a medium effect size gender difference in GG (*t* (99) = −2.87; *p* = .005; *d* = .635) and a small effect size in CG (*t* (99) = −2.05; *p* = .044; *d* = .453).

### Between-Group Analysis

Finally, comparisons by practice groups were performed for both ED and anxiety (see Table 3) using ANOVAs, followed, when necessary, by Bonferroni post-hoc testing. In total EDS-21, differences were found between BG-GG and BG-CG (*F (2, 231)* = 10.90; *p* = .001; *n*^*2*^ = .086), with BG presenting higher values. Testing separate ED criteria, group differences were found on “Continuance” between CG and GG, with CG presenting a higher mean value (*F (2, 231)* = 4.80; *p* = .009; *n*^*2*^ = .040), on “Tolerance,” where differences were found between CG-GG and BG-GG, with higher values for CG and BG, respectively (*F (2, 231)* = 12.07; *p* < .001; *n*^*2*^ = .095). Regarding “Reduction in other activities,” BG presented higher values compared with GG and CG (*F (2, 231)* = 13.02; *p* < .001; *n*^*2*^= .101). The same type of results was observed on “Time” (*F (2, 231)* = 13.35; *p* < .001; *n*^*2*^= .104). Finally, on “Interaction effects,” BG showed a higher value compared with GG (*F (2, 231)* = 6.75; *p* = .001; *n*^*2*^ = .055). All these differences were of a small effect size. Concerning ED prevalence among groups, a chi-square analysis by group was performed, and differences by group were found (*X*^2^ (4, *n* = 234) = 20.33, *p* < .001). Most practitioners in all groups were symptomatic (57.4% in GG, 75% in CG, and 67.3% in BG), but participants in BG presented a greater prevalence of an ED classification (18.4%), followed by CG (9.5%) and GG (5%). Group comparisons showed no group differences in participant levels of state or trait anxiety.

**Table 3.** Comparison of Variables Between Practice Group (*n* = 234).

	Gym (*n* = 101)			Cross training (*n* = 84)			Bodybuilding (*n* = 49)			F	*n* ^2^	Post Hoc
	*n* (%)	Mean (*SD*)	(CI 95%)	*n* (%)	Mean (*SD*)	(CI 95%)	*n* (%)	Mean (*SD*)	(CI 95%)			
EXD criteria												
Withdrawal effects		9.23 (4.48)	(3.00 - 18.00)		9.23 (6.67)	(3.00 - 18.00)		10.45 (4.91)	(3.00 - 18.00)	1.34	.011	
Continuance		6.59 (2.99)	(3.00 - 15.00)		8.18 (3.94)	(3.00 - 17.00)		7.88 (4.28)	(3.00 - 18.00)	4.80**	.040	2 > 1
Tolerance		9.51 (4 .20)	(3.00 - 18.00)		11.60 (3.60)	(4.00 - 18.00)		12.51 (3.56)	(4.00 - 18.00)	12.07***	.095	2 > 1
												3 > 1
Lack of control		7.24 (3.25)	(3.00 - 17.00)		8.19 (3.89)	(3.00 - 18.00)		8.18 (3.95)	(3.00 - 18.00)	1.98	.017	
Reduction on other activities		5.97 (3.10)	(3.00 - 15.00)		6.14 (2.81)	(3.00 - 14.00)		8.63 (3.79)	(3.00 - 18.00)	13.02***	.101	3 > 1
												3 > 2
Time		7.91 (3.83)	(3.00 - 18.00)		8.58 (3.78)	(3.00 - 17.00)		11.37 (4.21)	(3.00 - 18.00)	13.35***	.104	3 > 1
												3 > 2
Intention effects		6.18 (2.96)	(3.00 - 18.00)		7.06 (3.13)	(3.00 - 16.00)		8.18 (3.60)	(3.00 - 15.00)	6.75**	.055	3 > 1
EDS-21 total		52.63 (18.18)	(21.00 - 106.00)		58.98 (17.66)	(23.00 - 101.00)		67.20 (18.63)	(38.00 - 111.00)	10.90***	.086	3 > 1
												3 > 2
Classification for ED												
Asymptomatic	38 (37.60)			13 (15.50)			7 (14.30)					
Symptomatic	58 (57.40)			63 (75.00)			33 (67.30)					
Dependent	5 (5.00)			8 (9.50)			9 (18.40)					
State anxiety		38.81 (10.87)	(20.00 - 71.00)		38.51 (11.19)	(20.00 - 72.00)		39.98 (11.15)	(20.00 - 65.00)	.41	.004	
Trait anxiety		37.27 (9.92)	(20.00 - 68.00)		34.85 (11.06)	(20.00 - 75.00)		37.04 (10.02)	(20.00 - 65.00)	1.39	.012	

*Note*: CI 95%, confidence interval 95%; *SD* = standard deviation; ED = exercise dependence. *p < .05; **p < .01; ***p < .001.

## Discussion

In the present study, we aimed to characterize and report levels of ED and anxiety among a sample of community adults in Portugal who were participating in different groups of gym exercising (gym practitioners, cross trainers, and bodybuilders) but who were without access to facilities during the second period of the COVID-19 lockdown. Consistent with our hypothesis, practitioners of cross training showed a higher prevalence of ED symptoms than did practitioners of gym activities, but cross trainers reported a lower prevalence of ED symptoms than did bodybuilders. Males showed a higher prevalence of ED *symptoms* than females, but females showed higher values of exercise *dependence* than males in all groups. Thus, despite males’ higher prevalence of ED symptoms, our data reveal that females were more likely to develop dependence on exercise during the COVID-19 lockdown period and showed a higher prevalence of ED.

In prior research, bodybuilders and cross trainers have had higher ED values ​​when compared to other practice groups, including a higher prevalence than has been estimated for athletes generally (Marques et al., 2019). We found ED to have a higher prevalence among our cross training and body building participants during the COVID-19 pandemic than has been reported in previous studies (Lichtenstein & Jensen, 2016; Soler et al., 2013), while our GG group presented prevalence values ​​in line with values ​​reported in previous studies (Marques et al., 2019). Thus, some types of exercise practitioners, like bodybuilders and cross trainers are apt to experience more stress in a lockdown context, as gyms are where these practitioners associate time and dedication to their practice and strive for goals to be achieved.

When comparing the ED criteria between the groups, there were group differences in “Continuance”, “Tolerance”, “Reduction in other activities”, “Time” and “Intention effects”. Considering that our sample was composed of non-competing practitioners, these results are in line with other studies involving different groups of practitioners (Smith et al., 2010). In terms of gender analyses, females presented higher values of “Withdrawal” effects in GG and CG when considering analysis, but there were no gender differences between practice types. These results are consistent with previous research conducted before COVID-19 (Costa et al., 2013), in which females also showed higher values ​​of “Withdrawal” effects. These results seem to reveal that females were more likely to develop withdrawal symptoms in lockdown, except among bodybuilding practitioners. This greater risk for “withdrawal effects” for females may have derived from gender-based social factors (Abel et al., 2001), perhaps including activities and time demands that provided them less time for training outside of scheduled gym activities that were closed off to them during lockdown, while exercise for males may have been better socially supported (Hausenblas & Fallon, 2002). These factors may also be associated with men’s feelings of social disapproval when they reduce their practice (Masters & Lambert, 1989). On the other hand, higher “Withdrawal effects” may have also been associated with higher levels of anxiety among participants in GG and CG associated with their decreased access to practice.

In BG, no gender differences were found in “Withdrawal” effects and anxiety levels. This result may have been associated with higher levels of dedication and fulfilment of training tasks among BG practitioners, including females (Shilling & Bunsell, 2009), meaning that BG practitioners may have found ways to maintain their levels of practice. Direct surveys of these practitioners around this question in future research might resolve this puzzle.

Regarding “Continuance” (exercising despite injury or illness), there were no gender differences, but in practice group comparisons CG had the highest “Continuance” average and was significantly different from GG but not BG. Thus, cross trainers were predisposed to continue exercising even under the COVID-19 conditions, perhaps indicating some disruptive behavior in these circumstances, and a tendency to risk injury and/or health and well-being.

In terms of “Tolerance” (need for increasing amounts of exercise to achieve the desired results) there were only gender differences in BG, with males presenting a superior mean “tolerance” to females. Also, in BG, females showed higher values ​​when evaluating the intentions (often making more exercise than planned), and they showed an awareness that the time they dedicated to training often exceeded what would be necessary. An explanation for these results may be that these women’s pursuit of personal goals and achievements led them to depend on more time invested in practice. Across practice groups, BG had a higher “tolerance” average than GG but was not different from CG. Thus, both cross trainers and bodybuilders seemed to adopt behavior that reflected a value for high volumes of training to achieve results, and this trend was particularly pronounced in males.

For “Lack of control”, there were only gender differences in GG, with males showing higher values in terms of a persistent desire or unsuccessful effort to cut down or control exercise. These results seem to suggest that males had more difficulty managing time dedicated to exercise. In the practice group comparisons, there were no differences in “lack of control.” BG and CG may not feel need to control time in training as they are even predisposed to *increase* their practice time.

Regarding “Time” (i.g., spending too much time in exercise-related activities) and “Reduction in other activities” (i.g., occupational, social, or recreational activities are reduced or given up because of exercise) only the BG group was distinguished and differed from the others. Bodybuilders are predisposed to use much of their time in dedication to practice and even to sacrifice time for other activities such as leisure and social relationships. These results are in line with bodybuilding findings on other ED dimensions and may be partly explained by the fact that bodybuilding is a sport that requires a long period of dedication with a lifestyle and unique cultural system already part of in its practice (Hurst et al., 2000).

Cross training practitioners appear to have a behavior pattern that falls midway between regular exercisers and bodybuilders; but in some criteria, cross training participants in this study fell much closer to bodybuilding ED levels, with a higher ED prevalence than has been found in previous studies (Lichtenstein & Jensen, 2016). However, in their predisposition to train in a state of illness or injury, cross trainers presented the highest results, in line with a previous study (Lichtenstein & Jensen, 2016).

No group differences were found in participant anxiety levels during this pandemic context, suggesting that this variable depended more on gender than on the practice group. This finding seems to contrast with findings from Frontini et al. (2021) who found higher physical activity to be associated with lower anxiety. We found gender differences in anxiety in both GG and CG groups, just as Antunes et al. (2020) reported higher levels of state anxiety in women).

### Limitations and Future Directions

Among this study’s limitations were its cross-sectional design. A longitudinal research design is needed to make causal inferences regarding for the direction of the data relationships we revealed. Additionally, different ED protocols must be used to consolidate these results. We used a convenience sample, limiting generalizability to other populations. As we recruited participants over the internet, we did not survey or report our participants ethnicities, and the gender distribution was not balanced in GG. Future studies should expand and diversify participants and use objective observational and not just self-report measures to monitor anxiety and ED levels.

## Conclusions

In this survey of ED symptoms and classifications among three types of gym users during the COVID19 pandemic, we found greater ED among bodybuilders than cross-trainers and gym practitioners and higher anxiety among women than men. There were higher ED classifications among women than men and among bodybuilders than other practice groups. Both cross trainers and body builders experienced more ED symptoms than did gym practitioners generally and more than gym users in prior studies before the outset of COVID-19. We describe and discuss our findings in depth and outline directions for future research.

From a theoretical perspective, our results suggest that those advocating physical exercise (PE) as a means of promoting health and well-being should consider possible disruptive behaviors associated with pursuing PE intensely among specific practice groups like cross trainers and bodybuilders. Some practitioners may pursue exercise benefits without considering possible negative effects that may include ED that can be accentuated in the context of lockdowns and similar situations that may impede access to facilities. In addition, gender issues should be studied and theoretically framed, as there may be different gender-related different trends in behaviors and feelings associated with PE and ED.

From a practical perspective, professionals and researchers should be vigilant when resuming practice post-pandemic, because some groups of practitioners may have suffered from COVID-19 practice restrictions physically and psychologically. Prevalence ED should continue to be monitored and investigated, perhaps with comparison to data from this study. Practice modalities such as cross training that demand continuous exercise and considerable dedicated time should be promoted and monitored while considering the need to prevent ED symptoms that are often associated with eating disorders and other mental health concerns. While, typically, in gyms, sports facilities, and other contexts, exercise professionals have focused on the importance of engaging in more exercise, it is important in the context of COVID-19 lockdowns and associated isolations experienced currently and possibly into the future that professionals monitor their clients’ anxiety levels and search for compensatory time in training, while helping practitioners exercise in a healthy fashion.

## References

[bibr1-00315125221098326] Abel T. Graf N. Niemann S. (2001). Gender bias in the assessment of physical activity in population studies. Soz Präventivmed, 46(4), 268–272. 10.1007/BF01593182 11582854

[bibr2-00315125221098326] Alcaraz-Ibáñez M. Paterna A. Sicilia Á. Griffiths M. D. (2021). A systematic review and meta-analysis on the relationship between body dissatisfaction and morbid exercise behaviour. International Journal of Environmental Research and Public Health, 18(2), 1–21. 10.3390/ijerph18020585 PMC7827926 33445591

[bibr3-00315125221098326] American Psychiatric Association (2013). Diagnostic and statistical manual of mental disorders (5th ed.). 10.1176/appi.books.9780890425596

[bibr4-00315125221098326] Antunes R. Frontini R. Amaro N. Salvador R. Matos R. Morouço P. Rebelo-Gonçalves R. (2020). Exploring lifestyle habits, physical activity, anxiety and basic psychological needs in a sample of Portuguese adults during covid-19. International Journal of Environmental Research and Public Health, 17(12), 1–13. 10.3390/ijerph17124360 PMC7345948 32570737

[bibr5-00315125221098326] Berczik K. Szabó A. Griffiths M. D. Kurimay T. Kun B. Urbán R. Demetrovics Z. (2012). Exercise addiction: Symptoms, diagnosis, epidemiology, and etiology. Substance Use & Misuse, 47(4), 403–417. doi: 10.3109/10826084.2011.639120. 10.3109/10826084.2011.639120 22216780

[bibr32-00315125221098326] Cohen J. (1988). Statistical Power Analysis for the Behavioral Sciences (2nd ed.). Lawrence Erlbaum.

[bibr6-00315125221098326] Costa S. Hausenblas H. A. Oliva P. Cuzzocrea F. Larcan R. (2013). The role of age, gender, mood states and exercise frequency on exercise dependence. Journal of Behavioral Addictions, 2(4), 216–223. 10.1556/JBA.2.2013.014 25215203 PMC4154569

[bibr7-00315125221098326] Dasso N. A (2019). How is exercise different from physical activity? A concept analysis. Nursing Forum, 54(1), 45–52. 10.1111/nuf.12296 30332516

[bibr8-00315125221098326] Egorov A. Y. Szabo A. (2013). The exercise paradox: An interactional model for a clearer conceptualization of exercise addiction. Journal of Behavioral Addictions, 2(4), 199–208. . 10.1556/jba.2.2013.4.2 25215201 PMC4154576

[bibr9-00315125221098326] Frontini R. Rebelo-Gonçalves R. Amaro N. Salvador R. Matos R. Morouço P. Antunes R. (2021). The relationship between anxiety levels, sleep, and physical activity during COVID-19 lockdown: An exploratory study. Frontiers in Psychology, 12, 786. 10.3389/fpsyg.2021.659599 PMC8042226 33859601

[bibr10-00315125221098326] Hausenblas H. A. Fallon E. A. (2002). Relationship among body image, exercise behavior, and exercise dependence symptoms. International Journal of Eating Disorders, 32(2), 179–185. 10.1002/eat.10071 12210660

[bibr11-00315125221098326] Ho R. (2014). Handbook of univariate and multivariate data analysis with IBM SPSS (2nd ed.). Chapman and Hall/CRC. 10.1201/b15605

[bibr12-00315125221098326] Hurst R. Hale B. Smith D. Collins D. (2000). Exercise dependence, social physique anxiety, and social support in experienced and inexperienced bodybuilders and weightlifters. British Journal of Sports Medicine, 34(6), 431–435. 10.1136/bjsm.34.6.431. 11131230 PMC1724251

[bibr13-00315125221098326] Lichtenstein M. B. Jensen T. T. (2016). Exercise addiction in CrossFit: Prevalence and psychometric properties of the exercise addiction inventory. Addictive Behaviors Reports, 3, 33–37. 10.1016/j.abrep.2016.02.002 29531997 PMC5845980

[bibr14-00315125221098326] Lindwall M. Palmeira A. (2009). Factorial validity and invariance testing of the exercise dependence scale-revised in Swedish and Portuguese exercisers. Measurement in Physical Education and Exercise Science, 13(3), 166–179. 10.1080/10913670903050313

[bibr15-00315125221098326] Marques A. Peralta M. Sarmento H. Loureiro V. Gouveia É. R. Gaspar de Matos M. (2019). Prevalence of risk for exercise dependence: A systematic review. Sports Medicine, 49(2), 319–330. 10.1007/s40279-018-1011-4 30374944

[bibr16-00315125221098326] Masters S. Lambert J. (1989). On gender comparison and construct validity: an examination of the commitment to running Scale in a sample of marathon runners. Journal of Sport Behavior, 12(4), 196. (Ediç. Dec 1, 1989; Mobile, Ala).

[bibr19-00315125221098326] Shilling C. Bunsell T. (2009). The female bodybuilder as a gender outlaw. Qualitative Research in Sport and Exercise, 1(2), 141–159. 10.1080/19398440902909009

[bibr20-00315125221098326] Silva D. R. (2003). O inventário de estado-traço de ansiedade (STAI). In Gonçalves M. M. Simões M. R. Almeida L. S. Machado C. (Eds.), Avaliação Psicológica, instrumentos validados para a população portuguesa. Quarteto Editora.

[bibr21-00315125221098326] Smith D. Wright C. Winrow D. (2010). Exercise dependence and social physique anxiety in competitive and non-competitive runners. International Journal of Sport and Exercise Psychology, 8(1), 61–69. 10.1080/1612197X.2010.9671934

[bibr22-00315125221098326] Soler P. T. Fernandes H. M. Damasceno V. O. Novaes J. S. (2013). Vigorexy and levels of exercise dependence in gym goers and bodybuilders. Revista Brasileira de Medicina do Esporte, 19(5), 343–348. 10.1590/S1517-86922013000500009

[bibr23-00315125221098326] Spielberger C. D. Goruch R. L. Lushene R. E. Vagg P. R. Jacobs G. A. (1983). Manual for the state-trait inventory STAI (form Y). Mind Garden.

[bibr24-00315125221098326] Stapleton P. McIntyre T. Bannatyne A. (2016). Body image avoidance, body dissatisfaction, and eating pathology: Is there a difference between male gym users and non-gym users? American Journal of Men’s Health, 10(2), 100–109. 10.1177/1557988314556673 25389214

[bibr25-00315125221098326] U.S. Department of Health and Human Services (2018). Physical activity guidelines for Americans (2nd ed.). Accessed at: https://health.gov/our-work/nutrition-physical-activity/physical-activity-guidelines/current-guidelines

[bibr26-00315125221098326] Voelker D. K. Reel J. J. Greenleaf C. (2015). Weight status and body image perceptions in adolescents: Current perspectives. Adolescent Health, Medicine and Therapeutics, 6, 149–158. 10.2147/ahmt.s68344 26347007 PMC4554432

[bibr27-00315125221098326] Weinstein A. A. Koehmstedt C. Kop W. J. (2017). Mental health consequences of exercise withdrawal: A systematic review. General Hospital Psychiatry, 49, 11–18. 10.1016/j.genhosppsych.2017.06.001 28625704

[bibr28-00315125221098326] World Health Organization (2018). Global action plan on physical activity 2018-2030: More active people for a healthier world. Accessed at: https://www.who.int/ncds/prevention/physical-activity/global-action-plan-2018-2030/en/

[bibr29-00315125221098326] World Health Organization (2020a). Novel Coronavirus (2019-nCoV): Situation report – 10. https://www.who.int/docs/default-source/coronaviruse/situation-reports/20200130-sitrep-10-ncov.pdf?sfvrsn=d0b2e480_2 (Accessed 01 Nov 2021).

[bibr30-00315125221098326] World Health Organization (2020b). Novel Coronavirus (2019-nCoV): Situation report – 51. https://www.who.int/docs/default-source/coronaviruse/situation-reports/20200311-sitrep-51-covid-19.pdf?sfvrsn=1ba62e57_10 (Accessed 01 Nov 2021).

[bibr31-00315125221098326] World Medical Association (2013). World medical Association declaration of Helsinki ethical principles for medical research involving human subjects. JAMA: Journal of the American Medical Association, 310(20), 2191–2194. 10.1001/jama.2013.281053. 24141714

